# Creating a Sincere Sustainable Brand: The Application of Aristotle’s Rhetorical Theory to Green Brand Storytelling

**DOI:** 10.3389/fpsyg.2022.897281

**Published:** 2022-06-02

**Authors:** Chaohua Huang, Shaoshuang Zhuang, Ziyuan Li, Jingke Gao

**Affiliations:** ^1^Department of Business Administration, China University of Geosciences, Wuhan, China; ^2^School of Foreign Languages, Xinyang Normal University, Xinyang, China

**Keywords:** green brand storytelling, three means of persuasion, customer perceived value (CPV), perceived brand sincerity, brand trust, need for cognition (NFC)

## Abstract

As consumers become skeptical of green products, green brands may need to put trust-building on their business agenda. The study aims to use the rhetorical theory of Aristotle to examine the influence of a green brand story on perceived brand sincerity and brand trust. The study explores whether customer perceived value (CPV) mediates the effect between three means of persuasion used by a green brand story and perceived brand sincerity, and whether the need for cognition (NFC) plays a moderating role. A model is proposed and tested through three independent experiments in which participants were exposed to green brand stories and asked to complete a questionnaire. The results show that the green brand story with three means of persuasion has a more positive impact on perceived brand sincerity and brand trust than the green brand story without, and the impact is partially mediated by CPV. Besides, NFC moderates the effect: perceived brand sincerity of green brands improves with three means of the persuasion-laden story when NFC is relatively high. Specifically, the study reveals that pathos and ethos in a green brand story have positive effects on perceived brand sincerity through emotional value and social value, but the effect of logos is not identified. The findings contribute to the literature on brand storytelling, brand personality, and green marketing and have managerial implications for green brands to sustain a customer-brand relationship.

## Introduction

As people are becoming increasingly concerned about the effects of environmental issues, such as global warming on health, more non-green companies are also beginning to use green marketing to appeal to consumers, making deceptive green claims to build up a green image, which is known as greenwash (Chen et al., 2020). The expansive adoption of greenwash leads to consumer skepticism of green messages delivered by all firms, thus undermining the green market and endangering green purchases (Hamann and Kapelus, 2004).

To remove consumers’ distrust, companies could foster perceived authenticity through green brand storytelling (Huang and Guo, 2021). The strategic use of storytelling enables green brands to impress consumers and positively affects their behavioral intentions (Akgün et al., 2015), and rhetoric, a key element of brand stories, provides a collection of devices that writers can apply to persuade the reader to accept their viewpoints (Huang, 2010). However, research is scarce in investigating the effects of rhetoric in the context of brand storytelling. This is surprising, provided that rhetoric in the brand story is a powerful instrument to persuade potential customers to understand, interact, and finally make purchases (Ganassali and Matysiewicz, 2021).

Prior studies have reported that whether consumers trust a brand is dependent on both cognitive and emotional attributes of the brand (Delgado-Ballester, 2004). In other words, from the perspective of consumers, the credibility and reliability of a brand have a close tie to the personal value that the brand delivers to them (Napoli et al., 2016). As an important determinant of brand trust, perceived brand sincerity also results from a brand’s value-expressive attributes (Klipfel et al., 2014). While brand storytelling can create value for consumers (Ryu et al., 2019), therefore, the overall objective of the research is to exhibit that using rhetoric in a green brand story could strengthen perceived brand sincerity and consumers’ trust through customer perceived value (CPV). Specifically, it aims to answer the following research questions:

(1) What are the effects of three rhetorical appeals, logos, pathos, and ethos used by green brand stories on perceived brand sincerity?
(2) Whether CPV mediates the effect of three means of persuasion on perceived brand sincerity?
(3) Whether consumers’ perception of a brand’s sincerity through the green brand story will translate into brand trust?
(4) Whether there is an interaction effect between the need for cognition (NFC) and the three means of persuasion used by a green brand story?

So far, no study has applied Aristotle’s rhetorical theory to the green branding setting. The contributions of the present research to the current literature are three-fold: first, it identifies rhetorical strategies to craft a sincere and trustworthy green brand story, and it answers the call for more research on rhetoric in marketing (Soetaert and Rutten, 2019). Second, although existing literature on three means of persuasion abounds (Murthy and Gosal, 2016; Yang et al., 2018), few studies have rendered empirical investigations into how logos, pathos, and ethos influence the effect of advertising on creating value for consumers, which makes the study an innovative inquiry. Third, even though NFC has been used as a consistent moderator of the effects of rhetorical persuasion (Avramova et al., 2017), it has not been studied in terms of its role in moderating the relationship between brand story and perceived brand sincerity. Therefore, the study serves as the first attempt to measure this impact.

The rest of the article is structured in the following sections. Section “Literature Review and Hypotheses” presents the relevant theoretical background and develops the hypotheses and research model. Sections “Study 1,” “Study 2,” “Study 3,” demonstrate the methods used to test the proposed model and the results of each independent study. Finally, the article concludes with a discussion of research findings and implications, as well as limitations and future research directions in section “Discussion.”

## Literature Review and Hypotheses

### Green Brand Storytelling

Storytelling is very common in people’s daily life, serving as a tool for individuals to relate to each other and understand the world around them (Sanders and van Krieken, 2018). Practitioners in marketing, and especially in the advertising domain, employ storytelling as a powerful means to build a connection with and affect consumers (Rose, 2011). Advertisers use storytelling by depicting a chronological sequence of events in which characters engage with credible motivations, and it takes place in a specific setting that involves physical and social elements (Dessart, 2018). Storytelling is related to all aspects of brands. There are a variety of uses of storytelling by firms: organizational or brand stories, product stories, or consumer stories (Ryu et al., 2019). Brand stories could contain messages such as brand philosophy, vision statement, brand history, or how a brand is initiated (Kent, 2015). Brand stories provide an opportunity for marketers to model how consumers can derive meaning from the usage of their products (Singh and Sonnenburg, 2012). This is because stories allow brands to establish a framework in which brand identity is integrated, and consumers may connect with the brand within this framework (Ryu et al., 2019).

Research on brand storytelling abounds, but storytelling by green brands has rarely been studied. Huang and Guo (2021) believe that a green brand story should include a green brand’s commitment to the wellbeing of the society and a promise to protect the environment. A compelling and trustworthy green brand story, however, constitutes more than just green facts (Griffin et al., 2017; Ryu et al., 2019). Previous studies suggest that a compelling brand can approach storytelling in two ways: a contextual view and a structural view (Boje, 2014; Kent, 2015). The former concentrates on story contents, such as brand values, conflicts between characters, and historical connections (Boje, 2014), while the latter focuses on structural components by crafting a story with a first-person narrative voice, plot, and a chronology of the beginning, climax, and end (Kent, 2015; Ryu et al., 2019). Huang and Guo (2021) argue that infusing rhetoric into a green brand story will make the brand sound more compelling and authentic. They also verify that two rhetorical strategies, reversal and symbolism, help build brand trust. The present study thus follows this line of research in an attempt to gain an insight into green brand storytelling from a rhetorical perspective.

### The Effects of Aristotle’s Three Means of Persuasion on Perceived Brand Sincerity and Brand Trust

Aristotle defined rhetoric as “the ability, in each particular case, to see the available means of persuasion,” and identified three means of persuasion: logos (logical proof), ethos (ethical proof), and pathos (pathetic proof) (Borchers, 2005). Logos concerns itself with logical reasoning in statements made by speakers. Ethos refers to words that speakers leverage on to manifest their credible character, goodwill, or attitude. Pathos appeals to the emotions or feelings of the audience (Bonet and Sauquet, 2010). This would mean that if common ground is sought between two parties who share different viewpoints, logos, ethos, and pathos are essential for one to persuade another (Panigyrakis et al., 2020). Aristotle’s rhetorical theory has been applied to a myriad of studies in fields, such as advertising, industrial communication, and e-commerce marketing (Murthy and Gosal, 2016; Yang et al., 2018). The focus of rhetorical analysis shifts from criticizing long-standing debates in marketing (Phillips and McQuarrie, 2004) to examining current marketing practices (Panigyrakis et al., 2020). Increasingly, studies in this area have revealed how rhetorical figures, well-intended language, and right actions assist the effectiveness of marketing efforts (Yang et al., 2018; Panigyrakis et al., 2020).

Brand personality refers to a group of personality attributes that consumers associate with a brand (Aaker, 1997). A brand’s personality embodies consumers’ understanding of what a brand means to them (Schmitt, 2012). Brand sincerity is a dimension of brand personality, which is defined by Aaker (1997) as the quality of being honest, wholesome, down-to-earth, cheerful, and genuine. A myriad of research has studied brand sincerity in different ways. Some researchers have investigated the effect of sincerity on the social media/e-commerce industry and reported that social media celebrities who have a high level of perceived sincerity are more likely to establish a rapport with customers as opposed to celebrities having a low level of perceived sincerity (Lee and Eastin, 2020). Sincerity has also been researched in the sports brand domain where Braunstein and Ross (2010) developed a context-specific instrument for perceived sincerity: *sincere sports brand*. In addition, another realm where brand sincerity is emphasized to have a positive impact is corporate social responsibility (CSR), where it has been tested that firms’ engagement in CSR initiatives entails brand sincerity (Mandal et al., 2021). To the best of our knowledge, however, there is no research concerning the impact of green brand storytelling on perceived sincerity. Therefore, this study specifically concentrates on analyzing this impact.

Brand storytelling creates value for consumers as it is associated with their needs (Ryu et al., 2019). Logos, pathos, and ethos all speak powerfully to the needs of the target audience (Borchers, 2005). Thus, applying the three means of persuasion to brand storytelling is conducive to communicating with consumers and addressing their concerns. As perceived brand sincerity is the product of a brand’s value-expressive attributes (Klipfel et al., 2014), it is argued that the use of rhetoric to foreground the positive brand attributes in a green brand story can translate into perceived brand sincerity. Based on the literature, the study proposes the first hypothesis:

H1: A green brand story with three means of persuasion is more likely to strengthen perceived brand sincerity than a green brand story without.

Torto (2020) suggests that rhetorical persuasion can be achieved when an ad text writer is recognized as reliable, when strong emotional responses are induced among consumers, and when brand facts are communicated. In the study, the logical proof is represented by inductive reasoning or deductive reasoning; the pathetic proof is represented by emotive words; the ethical proof is represented by adjectives that emphasize the good qualities and attributes of the product advertised (Torto, 2020). In the same light, Chu et al. (2014) found that logos, pathos, and ethos play a significant role in enhancing the persuasiveness of e-commerce product pages, indicating that the visual design, logical structure, playfulness, empathy, use of metaphors, and simple and concise language are representations of the three persuasive cues. Among them, empathy is defined as the identification process whereby a brand aligns with target consumers’ interests and communicates a sense that relates to their values and desires (Higgins and Walker, 2012). In the green brand storytelling setting, empathy means an emphasis on the functional benefits of green attributes. For instance, rather than highlighting the general environmental benefit, a green brand story can associate a green purchase with reduced penalty risk, reduced electricity bill, or reduced health hazards (Pickett-Baker and Ozaki, 2008).

Logos is the basic cue that enables consumers to make a fact-based assessment by evaluating the textual elements presented in a green brand story, which demonstrates the honesty of a green brand. Pathos aligns with target consumers’ interests and communicates a sense that is concerned with their values and desires (Higgins and Walker, 2012), thus conveying cheerfulness to consumers. Ethos can be seen as a green brand’s credibility, reliability, and assurance, the degree to which consumers acquire information about the brand’s credibility from the expert knowledge and green business certifications provided in the story (Higgins and Walker, 2012; Chu et al., 2014). In other words, ethos is pivotal to a green brand’s genuineness. When the three means of persuasion act concurrently, the effectiveness of communication becomes stronger (Yang et al., 2018). Therefore, the study proposes that:

H2: A green brand story with logos will strengthen perceived brand sincerity.

H3: A green brand story with pathos will strengthen perceived brand sincerity.

H4: A green brand story with ethos will strengthen perceived brand sincerity.

Trust is recognized as “the expectation that arises within a community of regular, honest, and cooperative behavior, based on commonly shared norms, on the part of the members of the community” (Fukuyama, 1995, p. 26). Trust in a brand falls into two parts—confidence in the perceived reliability (cognitive trust) and perceived credibility stemming from consumers’ expectations, values, and feelings (affective trust) (Servera-Francés and Piqueras-Tomás, 2019). Previous research has reported perceived sincerity to be one of the strongest antecedents of consumer trust, and compared with other brand personalities, perceived brand sincerity is the only dimension pertinent to building brand trust (Perepelkin and Di Zhang, 2014). Besides, sincerity can evoke inferences about the trustworthiness of a spokesperson (Puzakova et al., 2015). Therefore, the study proposes the fifth hypothesis:

H5: Perceived brand sincerity has a positive effect on brand trust.

### The Mediating Role of Customer Perceived Value

Customer perceived value is conceptualized by Zeithaml (1988) as a consequence of a consumer’s tradeoff of benefits and costs in the purchase of a product. Such a notion treats “value” as a unidimensional construct that emphasizes the cognitive and economic dimensions of customers’ value perceptions (Zauner et al., 2015). However, narrowly viewing CPV in this way excludes the possibility of unraveling other features of value derived from consumption experiences (Sanchez-Fernandez and Iniesta-Bonillo, 2007). Some authors have suggested that CPV is a multidimensional concept that embraces several kinds of value simultaneously (Sheth et al., 1991; Sweeney and Soutar, 2001). In addition to cognitive and economic features, emotional, social, symbolic, hedonic, and aesthetic dimensions are also relevant to CPV (Sheth et al., 1991). The study decides to continue along with this standpoint and view CPV as multi-faceted as Sweeney and Soutar (2001) point out that “a more sophisticated measure is needed to understand how consumers value products and services” (p. 201).

According to the above argument, the study considers CPV to be a function of three dimensions, functional value, emotional value, and social value. Functional value refers to the utility a market offering creates through its expected quality (Sweeney and Soutar, 2001). Emotional value refers to the utility an offering provides through feelings and affective states, while social value mainly addresses the utility originating from the enhanced social self-concept of an individual (Sweeney and Soutar, 2001).

Although CPV has been a hot research topic in many areas, it has not been researched regarding green brand storytelling. It is established that consumer-targeted CSR actions and service quality can increase CPV (Yarimoglu, 2017; Servera-Francés and Piqueras-Tomás, 2019), and several lines of evidence show that CPV has a significant effect on consumers’ trust (Oghuma et al., 2016; Luo and Ye, 2019). Green brand storytelling allows brands to fulfill both the social and environmental facets of consumer needs. Value, therefore, is represented in brand stories by sustainable business practices. Logos can increase the quality of information delivered to consumers by using concise and logical language (Chu et al., 2014), which has functional value. Pathos, on the other hand, with the aim to entertain consumers through emotion-laden words, is relevant to emotional value. Ethos that places emphasis on building brand credibility and reputation is a determining factor of social value (Fiol et al., 2009). These values in turn can improve perceived brand sincerity (Servera-Francés and Piqueras-Tomás, 2019; Mandal et al., 2021). According to the preceding discussion, the study proposed the following hypotheses:

H6: The effect of a green brand story with three means of persuasion on perceived brand sincerity is mediated by CPV.

H7: The effect of a green brand story with logos on perceived brand sincerity is mediated by functional value.

H8: The effect of a green brand story with pathos on perceived brand sincerity is mediated by emotional value.

H9: The effect of a green brand story with ethos on perceived brand sincerity is mediated by social value.

### The Moderating Role of the Need for Cognition

The NFC is theorized by Cohen et al. (1955) as “a need to structure relevant situations in meaningful integrated ways” (p. 291). It is reflective of a person’s willingness to enjoy and engage in processing information and making sense of things. As such, NFC has always been used as a moderator of the effects of rhetorical persuasion (Avramova et al., 2017). Therefore, the study taps into NFC to reveal the effects of NFC on the rhetoric used in green brand storytelling. Individuals who score high in NFC tend to be more rational and think more deeply than those who score low in NFC (Ott et al., 2016). Due to their higher tendency to engage in cognitive endeavors, they will be more susceptible to the quality of persuasive arguments. Their cognitive processing mainly centers around brand strategy, brand personality, product features, and other clues, which they analyze in more detail and more mindfully when evaluating a brand (Haugtvedt et al., 1992). Therefore, their evaluation will be more comprehensive. The brand story integrating three means of persuasion demands more cognitive effort as it encapsulates various information about the brand, which may hamper the information processing of individuals that are low in NFC. In contrast, individuals with high NFC are more likely to expend efforts in examining the narrative contents in a brand story (Wheeler et al., 1999) and thus are more likely to be immersed in the story (Green et al., 2008) and perceive the brand sincerity. Thus, the study puts forward the following hypotheses:

H10: Need for cognition moderates the relation between a green brand story with three means of persuasion and perceived brand sincerity.

H10(a): For individuals with high NFC, the green brand story with three means of persuasion can significantly affect perceived brand sincerity.

H10(b): For individuals with low NFC, the green brand story with three means of persuasion does not significantly affect perceived brand sincerity.

**FIGURE 1:**
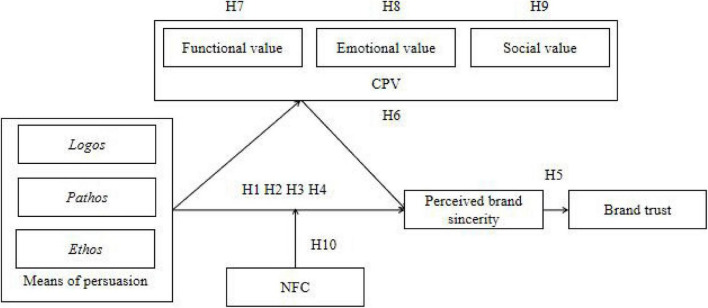
Research model.

Based on the literature above, the study proposed the research model, as shown in Figure 1.

## Study 1

The first study investigates whether a green brand story loaded with three means of persuasion has a more positive influence on participant’s perceived brand sincerity and brand trust than a green brand story without the three means of persuasion (H1 and H5), and whether CPV mediates the effect of the rhetoric-laden story on perceived brand sincerity (H6).

### Stimulus Materials and Experimental Procedure

The study crafted two fictitious green brand stories to control for participants who had prior experiences of purchase and use with real brands (Carnevale et al., 2018) (as shown in Supplementary Appendix). The study adopted the salient dimensions from the framework proposed by Chu et al. (2014) based on the studies of Yang et al. (2018) and Torto (2020). Four professors in marketing and a marketing manager were invited to group those dimensions within the three rhetorical appeals. After discussion, we identified nine story design features for eight salient factors closely related to a green brand story. Table 1 demonstrates the dimensions of operating elements in the tested green brand stories. To control for the effect of other experimental factors, the brand name and the storyline of the two brand stories are made the same except for the differences listed in the Table 1. Specifically, for pathos, the green brand story with three means of persuasion used a “growth” metaphor, which describes the development of a company, a “strength” pun, and emphasized the functional benefit that the product poses no threat to children (as shown in Supplementary Appendix).

**TABLE 1:** List of manipulated factors in a green brand story.

Means of persuasion	Salient dimensions	Content features	Operational levels
Logos	Story format	Story style	Concise/Verbose
	Brand facts	The use of concrete numbers	Present/Absent
Pathos	Entertainment	Use humor	Present/Absent
	Tangibility	Use metaphor	Present/Absent
	Empathy	Provide information on functional benefits of products	Present/Absent
Ethos	Credibility	Expert knowledge	Present/Absent
		The use of adjectives to highlight the product attributes	Present/Absent
	Assurance	Provide information about sustainable certification	Present/Absent
	Reliability	Provide contact information such as e-mail, phone number, and company address	Present/Absent

A pretest was conducted to ensure the appropriateness of using the factors in a green brand story. We used the Narrative Structure Coding Scale of Escalas (2004) to conduct a manipulation check to ensure that the two green brand stories are well-structured, preventing the effect of rhetoric from being influenced by the structural components of a story. In the main test, an intergroup design for a single factor is recruited (the green brand story with rhetoric vs. the green brand story without rhetoric). After being randomly exposed to a story, participants were asked to finish a questionnaire. The questionnaire includes three items of brand sincerity (honest, down-to-earth, and cheerful) adapted from Aaker (1997), three items (trustworthy, credible, and authentic) from the brand trust scale of Hsu et al. (2011), and nine items of CPV adapted from Sweeney and Soutar (2001). A seven-point Likert scale was adopted for measuring the variables. Besides, a pilot study was conducted with 22 undergraduate students who were interviewed about ease of comprehension. Some modifications were made according to their feedback. In total, 289 participants were involved in the study. Incomplete and inconsistent questionnaires and those that were completed in less than 1 min were excluded, leaving 251 valid questionnaires, filled in by 115 women (45.6%) and 137 men (54.4%). The sample size of the rhetoric story group is 128, while the sample size of the non-rhetoric story group is 123. The demographic information of participants is displayed in Table 2.

**TABLE 2:** Demographic profiles (*N* = 251).

Measure	Items	Frequency	Percentage
Gender	Men	136	54.4
	Women	115	45.6
Age	18–25	127	50.8
	26–35	93	36.9
	36–45	22	8.7
	>45	9	3.6
Education	High school or below	55	21.8
	Bachelor degree	117	46.8
	Graduate degree	79	31.4
Job	Student	118	47.2
	Office worker	73	29.0
	Firm owner	16	6.3
	Freelancer	44	17.5

### Results

For the manipulation check, the study tested the reliability of six items on the Narrative Structure Coding Scale. The result demonstrates that Cronbach’s alpha value (0.850) exceeds the 0.70 cutoff (Nunnally, 1978) value, suggesting the two stories are well-structured.

#### Perceived Brand Sincerity

The results show that the three means of persuasion in the green brand story have a significant impact on perceived brand sincerity and that the green brand story with the rhetoric is more likely to induce participants’ brand sincerity perception than the story without (*M*_story  with  rhetoric_ = 5.24, SD = 0.95, *M*_story  without  rhetoric_ = 4.11, SD = 1.23, *F* = 65.452, *p* < 0.001, *f* = 0.513), thus confirming H1.

#### Brand Trust

The results show that the perceived brand sincerity will significantly affect brand trust and that the story with rhetoric leads to greater brand trust (*M*_story  with  rhetoric_ = 5.63, SD = 1.20, *M*_story  without  rhetoric_ = 4.67, SD = 1.38, *F* = 34.61, *p* < 0.001, *f* = 0.373). Therefore, H5 is supported. Table 3 shows the result.

**TABLE 3:** Differences in the influence of the three means of persuasion in green brand stories on perceived brand sincerity and brand trust.

	Story loaded with rhetoric (M ± SD)	Story without rhetoric (M ± SD)	*F*	Cohen’s *f*
Perceived brand sincerity	5.24 ± 0.95	4.11 ± 1.23	65.452**	0.513
Brand trust	5.63 ± 1.20	4.67 ± 1.38	34.610**	0.373

**p < 0.01.

#### Mediating Effect of Customer Perceived Value

**FIGURE 2:**
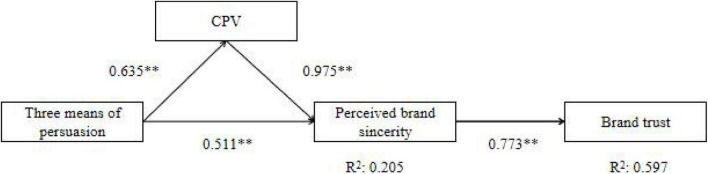
Results of the hypothesized model. **p < 0.01.

The study analyzed the mediating role of CPV through bootstrapping (Hayes and Scharkow, 2013). The results show that CPV only partially mediates the influence of the three means of persuasion in a green brand story on perceived brand sincerity (95% confidence interval (*CI*) β = 1.129, *CI* = 0.856–1.403). The green brand story with the rhetoric will affect the CPV (Path 1: β = 0.635, *CI* = 0.392–0.878), thus affecting perceived brand sincerity (Path 2: β = 0.975 *CI* = 0.905–1.045). After controlling the mediating factor, the direct effect of the green brand story with rhetoric on perceived brand sincerity is also significant (direct effect β = 0.511, *CI* = 0.366–0.655), which indicates that CPV partially mediates the relationship between rhetoric and perceived brand sincerity. The results support H6. Figure 2 summarizes the results of this study.

## Study 2

The study examines whether the three rhetorical appeals, logos, pathos, and ethos, in a green brand story have positive influences on brand sincerity (H2–H4) and whether their influences are, respectively, mediated by functional value, emotional value, and social value (H7–H9).

### Sampling and Survey Instruments

Data were collected based on an online survey. The study contracted a professional Chinese research company, Wenjuan Pro, to conduct the investigation. A total number of 256 participants’ responses were received. Incomplete or apparently random responses were excluded, leaving 227 valid samples, which exceeds the minimum 200 observations suggested by Marsh and Bailey (1991).

The survey instruments were either developed on the basis of measurement items validated by previous studies or derived from Aristotle’s definitions of persuasive means to fit the context of the present survey. The questionnaire applied for collecting data involved scales to measure constructs of the theoretical model. The questionnaire items were reviewed and assessed by four academic experts and one marketing practitioner to ensure the validity of content. Besides, a pilot study was conducted with 20 college students who were interviewed about the ease of comprehension. Some modifications were made according to their feedback. All items were gauged using a seven-point Likert-style scale, ranging from strongly disagree (1) to strongly agree (7). In particular, the construct of perceived brand sincerity was measured with three items developed on the basis of measurements from Aaker (1997), while CPV was measured with nine items derived from Sweeney and Soutar (2001). The three items of logos were adapted from Aristotle’s definitions of logos as well as from the study of Chu et al. (2014). Three items for pathos were adapted and reworded in accordance with the empathy scales of Davis (1994), which foreground perspective taking and empathic concern. Another item was adapted from Chiu et al.’s (2012) scale to reflect the effect of humor in a brand story. To operationalize ethos, items were adapted from Yang et al. (2018), which focused on the credibility of the story.

### Reliability and Validity of Measures

In this study, data were analyzed using AMOS 23.0 and SPSS 22.0. First, the results exhibit good model fit [*X*^2^/df (2.06) is less than 3; non-normed fit index (NNFI) (0.924), comparative fit index (CFI) (0.940), Tucker–Lewis index (TLI) (0.924), and incremental fit index (IFI) (0.941) are greater than 0.9; root mean square error of approximation (RMSEA) (0.069) is less than 0.08] between the factor structure and the data (Tabachnick and Fidell, 2013). The reliability of the proposed seven-factor measurement model composed of logos (LO), pathos (PA), ethos (ET), functional value (FV), emotional value (EV), social value (SV), and perceived brand sincerity (PBS) was tested with Cronbach’s alpha. As shown in Table 4, all the seven latent constructs in the study have good reliability with the value of α ranging from 0.85 to 0.88, which exceeds the standard level of 0.7 (Nunnally, 1978). It is also observable from Table 4 that each construct is significantly correlated to each other as correlations between them are lower than the standard 0.85 (Kline and Santor, 1999).

**TABLE 4:** Cronbach’s alpha values and correlations.

	α	M	SD	LO	PA	ET	FV	EV	SV	PBS
LO	0.86	5.06	0.80	1						
PA	0.86	5.03	0.93	0.51**	1					
ET	0.86	5.05	0.96	0.60**	0.56**	1				
FV	0.88	4.47	1.15	0.38**	0.45**	0.43**	1			
EV	0.88	5.55	1.16	0.40**	0.47**	0.44**	0.38**	1		
SV	0.85	5.15	0.88	0.61**	0.64**	0.61**	0.61**	0.59**	1	
PBS	0.86	5.03	0.98	0.57**	0.59**	0.64**	0.49**	0.42**	0.65**	1

**p < 0.01.

Second, according to the results shown in Table 5, all standardized factor loadings are statistically significant spanning from 0.703 to 0.855, exceeding the 0.7 cutoff value that indicates adequate convergent validity. Besides, the average variance extracted (AVE) of all constructs fall above 0.5, ranging from 0.570 for PA to 0.693 for EV. Concomitantly, all values of composite reliability (CR) are higher than 0.7, suggesting adequate reliability (Hair et al., 2006).

**TABLE 5:** Measured items.

Constructs		Factor loadings	AVE	CR
Logos (Story logic)	The story uses numbers to support its viewpoints	0.801	0.599	0.817
	The story is concise	0.703		
	The story highlights the greenness of the product	0.812		
Pathos (Emotional bonding)	The pun used in the last sentence is playful	0.734	0.570	0.798
	I can emphasize with the situation described in the story	0.716		
	I am touched by what I see happen in the story	0.811		
Ethos (Credibility)	The product knowledge offered by the story is reliable	0.799	0.638	0.841
	The product attributes are positive	0.796		
	I believe the product is effective and eco-friendly	0.801		
FV	The product has good quality	0.735	0.617	0.828
	The product would, in functional terms, perform well	0.837		
	The green product is useful	0.780		
EV	I want to have the product of this brand	0.841	0.693	0.872
	Buying this product will make me feel good	0.855		
	The green brand is one with which I feel satisfied	0.802		
SV	Buying this product influences the image that others have of me	0.737	0.579	0.805
	Buying this product would create a favorable perception of me among other people	0.754		
	The green brand likes a credible person to whom I can relate	0.792		
PBS	The green brand is honest	0.776	0.583	0.807
	The green brand is down-to-earth	0.740		
	The green brand is cheerful	0.775		

### Hypothesis Testing Results

The structural equation modeling (SEM) approach was harnessed to examine the main effect of the proposed model. First, the fit indices (X^2^/df = 1.85, NNFI = 0.959, CFI = 0.97, TLI = 0.959, IFI = 0.97, and RMSEA = 0.061) indicate that the model has a satisfactory fit (Tabachnick and Fidell, 2013). After obtaining an acceptable fit between the structural model and the data, the study then tested H2–H4. The coefficients, *t*-value, and overall explanatory power (*R*^2^ value) are displayed in Table 6. Not as expected, the results only offered support for H3–H4, whereas H2 was rejected. More specifically, perceived brand sincerity was significantly improved by pathos (β = 0.351, *t* = 3.160, *p* = 0.002) and ethos (β = 0.472, *t* = 4.247, *p* < 0.001).

**TABLE 6:** Results for research hypotheses.

Hypotheses	Standardized regression weights	C.R. (*t*-value)	*p*-value	Hypothesis status
H2: Logos→PBS	0.091	0.799	0.424	Not supported
H3: Pathos→PBS	0.438	3.160	**	Supported
H4: Ethos→PBS	0.904	4.247	***	Supported

*PBS R^2^: 0.694.*

**p < 0.01 and ***p < 0.001.

**FIGURE 3:**
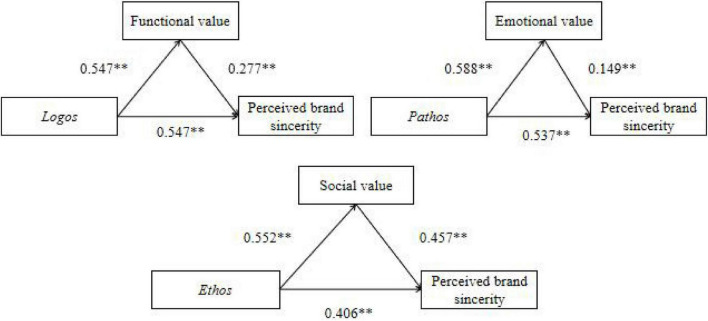
Mediation analysis. **p < 0.01.

A mediation analysis was then performed to check for the association between three means of persuasion and perceived brand sincerity mediated by three dimensions of CPV. A bias-corrected bootstrap analysis was conducted to test the indirect effect. Following the recommendation of Preacher and Hayes (2008), the study rendered repeated extractions of a bootstrapped sample of 5,000 from the data to scrutinize the significance of the estimates with the associated 95% *CI*. The results exhibited that functional value, emotional value, and social value only partially mediate the relationship between logos, pathos, ethos, and perceived brand sincerity, with the indirect effect excluding zero (β = 0.698, *CI* = 0.068–0.182; β = 0.625, *CI* = 0.024–0.143; β = 0.658, *CI* = 0.164–0.334). Similarly, the direct effect of pathos on perceived brand sincerity is still significant when CPV is introduced into the model. Therefore, H7, H8, and H9 are supported. Figure 3 delineates the standardized path coefficients on the structural model.

## Study 3

### Research Design and Participants

A 2 (rhetoric-loaded brand story vs. non-rhetoric-loaded brand story) × 2 (high NFC vs. low NFC) between-subjects design was used to examine whether the effect of a rhetoric-loaded green brand story on consumer’s perceived brand sincerity is moderated by NFC (H4). Data were collected based on an online survey. Participants were randomly exposed to each of the two green brand stories and asked to complete a questionnaire. In total, 117 participants participated in the experiment.

### Stimulus Materials and Measures

To avoid the experience with existing brands from affecting the results of the study, two fictitious brand stories were used as experimental stimuli. The story structure, brand name, and protagonists of the two stories were made the same except for the differences listed in Table 1. Specifically, the green brand story with three means of persuasion used a structural metaphor (SUCCESS IS PATH) that emphasizes sports spirit, an “irritate” and a “company” pun to induce a humorous effect, implying the functional benefit that the product will not cause irritation to skin (as shown in Supplementary Appendix).

In a pretest, Escalas (2004) Narrative Structure Coding Scale was used to make sure that each story was written with a complete story structure. All items were gauged using a seven-point Likert-style scale ranging from strongly disagree (1) to strongly agree (7). Specifically, the construct of perceived brand sincerity was measured with three items developed on the basis of measurements from Aaker (1997). The moderating effect of NFC was measured based on the 18-item NFC scale provided by Cacioppo and Petty (1982). However, owing to the unreliability of using both positive and negative items of the scale (Benson and Hocevar, 1985), only the nine positive items were adopted in this measure. The participants were asked to rate NFC on a 7-point Likert-type scale (1 = extremely uncharacteristic of me and 7 = extremely characteristic of me).

### Results

For the manipulation check, the study tested the reliability of six items on the Narrative Structure Coding Scale. The result demonstrates that Cronbach’s alpha value (0.831) exceeds the 0.70 cutoff value (Nunnally, 1978), suggesting that the two stories are well-structured.

The numerical interval of the NFC is [18,127], and the median is 83.73. Participants whose scores were above the median were assigned to the high NFC group, while those below the median were assigned to the low NFC group. For statistical purposes, this study encoded high NFC as 1 and low NFC as 0, the green brand story with three means of persuasion as 1, and the green brand story without three means of persuasion as 0.

#### Main Effect Analysis

The results show that the main effect on perceived brand sincerity of the green brand story with three means of persuasion is significant (*F* = 37.98, *p* < 0.001). H1 is supported again. The marginal main effect of an individual’s NFC on perceived brand sincerity is also significant (*F* = 14.48, *p* < 0.001).

#### Interaction Analysis

Taking perceived brand sincerity as the dependent variable, we carried out a 2 (rhetoric-loaded brand story vs. non-rhetoric-loaded brand story) × 2 (high NFC vs. low NFC) analysis of variance, and the results show that the interaction of the green brand story with three means of persuasion and NFC significantly affects perceived brand sincerity (*F* = 44.28, *p* < 0.001).

#### Simple Effect Analysis

**FIGURE 4:**
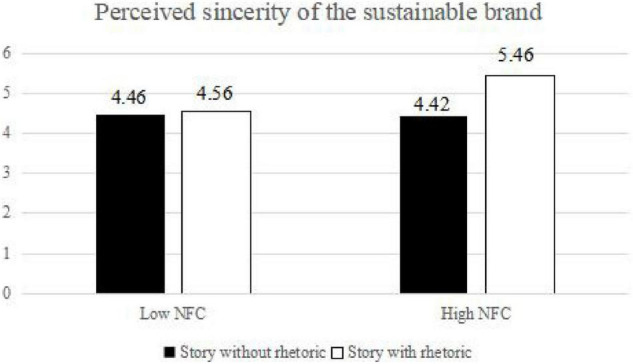
The moderating role of the need for cognition (NFC).

To verify that the main effect, the marginal main effect, and the interaction effect are significant, the participants were assigned to a high NFC group and a low NFC group according to the median (83.73) through a 2 (rhetoric-loaded brand story vs. non-rhetoric-loaded brand story) × 2 (high NFC vs. low NFC) grouping. A simple effect analysis was performed in the experiment. The sample size of each group is as follows: *N*_story  with  rhetoric,  high  NFC_ = 27; *N*_story  without  rhetoric,  high  NFC_ = 27; *N*_story  with  rhetoric,  low  NFC_ = 28; and *N*_story  without  rhetoric,  low  NFC_ = 28. For individuals with low NFC, the green brand story with three means of persuasion has no significant impact on perceived brand sincerity (*M*_story  with  rhetoric_ = 4.56, SD = 0.25, *M*_story  without  rhetoric_ = 4.46, SD = 0.34, d = 0.31; *F* = 1.40, *p* > 0.05). For individuals with high NFC, the green brand story with three means of persuasion has a significant impact on perceived brand sincerity (*M*_story  with  rhetoric_ = 5.46, SD = 0.29, *M*_story  without  rhetoric_ = 4.42, SD = 0.51, *d* = 2.53; *F* = 7.96, *p* < 0.001) (as shown in Figure 4). Therefore, H10 is supported.

## Discussion

### Theoretical Implications

Brand sincerity and CPV are among the most popular topics in marketing studies, but there is a dearth of research that associates them with brand storytelling, which makes it an innovative inquiry. The results of this study demonstrated that the three means of persuasion in a green brand story have a significantly positive effect on brand sincerity and brand trust, reflecting that the strategic use of language is beneficial in building the brand-consumer relationship, thus contributing to the literature by extending the brand storytelling research to the area of rhetoric. Although some recent studies started to consider attempts made by brands on sustainable marketing, very few of them have taken a look at the use of rhetoric in green branding. Further, prior studies have utilized some other theories to explain how brand storytelling works, such as narrative transportation (Ryu et al., 2019; Huang and Guo, 2021), but to the author’s knowledge, there is no empirical study investigating the role of CPV in mediating the effect between brand story and brand personality. The study serves as an initial attempt to explore this influence.

The study put forward a theoretical model, based on Aristotle’s rhetorical theory, to measure the impact of the three means of persuasion, logos, pathos, and ethos, on brand sincerity. According to prior literature that suggest these persuasive techniques represent the relevant attributes that consumers take into consideration when evaluating a product or service (Chu et al., 2014; Yang et al., 2018), the study also proved that they could be effective in writing skills in crafting a green brand story. To be more specific, conciseness, concrete numbers, humor, metaphor, green business certifications, and contact address in a brand story could be helpful to establish a sincere brand image in the mind of consumers, which is consistent with Chiu et al.’s (2012) finding that conciseness and humor are vital story components to affect brand attitude. The study findings also illuminated that NFC moderates the effect of a green brand story on brand sincerity. It is implied that the use of the three means of persuasion in a brand story may be more appropriate for those who tend to think mindfully.

The findings of Study 2 show two rhetorical appeals used by a green brand story, pathos and ethos, have a significantly positive effect on perceived brand sincerity. The term pathos is understood by the study as an emotional bonding formed between brands and consumers, which relates to the empathic concern and humorous effect of a green brand story. The results thus are consistent with prior literature that humor and empathy in brand stories are positively associated with brand attitude (Chiu et al., 2012; Yu and Chang, 2013). Ethos is interpreted by the study as the extent to which male consumers abstract information about the narrator’s credibility from the green brand story. The results, therefore, validate the findings of previous studies that credibility is positively related to brand attitude (Hayes and Carr, 2015). However, the study findings reveal that logos fail to exert a significant influence on perceived brand sincerity. This implies that consumers’ perception of green brand sincerity is not determined by the logical reasoning of a story or how the story is formatted but is dependent on the emotional appeal and perceived credibility of the story. It is possible that these elements are universal persuasive cues in traditional brand stories, and their effect on brand sincerity becomes less salient when used in a sustainable brand story. It is also possible that the items weighing logos are not exhaustive as this construct was self-developed based on previous studies and Aristotle’s definition. Future studies could improve the measures and repeat the research.

### Managerial Implications

The main managerial implication is that, for green firms, managers are provided with insightful ideas to sustain the brand-consumer relationship. Recruiting rhetoric in green brand storytelling can narrow down the psychological distance between consumers and the brand (Huang and Guo, 2021). In particular, the message conveyed to consumers should, on the one hand, generate value for them, and, on the other hand, align with their functional and mental needs (Lin and Zhou, 2020). For instance, it is inadequate to emphasize the greenness of the brand, but an elaboration on what green business certifications have been obtained, what kind of expert advice is provided, what positive emotions can be induced among consumers, or what specific numbers are presented, among others, is more relevant. As distrust in green marketing is prevailing, and consumers are inclined to develop a stereotype of a green brand (Chen et al., 2020), it is important to use storytelling to differentiate the brand as much as possible. Consumers will transfer their trust toward a green brand that conveys sincerity in brand stories. Therefore, green firms should take advantage of brand storytelling in a manner that creates value for consumers.

Second, green brand storytelling is not only about the construction of a story with necessary structural elements but also, in fact, about the incorporation of contents that trigger the resonance of consumers. Thus, it is suggested that green firms should give close attention to the quality of the message they send to consumers through the means of brand storytelling. A famous online celebrity in China, Li Ziqi, is very adept at telling the story of her products to the audience. Traces of functional value, emotional value, and social value can all be found in her videos. She communicated with her audience through traditional Chinese culture in a very logical and affective fashion. Her pro-environmental behavior is represented in her making the best use of every ingredient of cooking to avoid waste and recycled use of raw materials, moving consumers into believing that the product is organic and green. Further, comments from her videos suggest that these stories can remind people of their childhood and good old days, which implies that many people can empathize with her daily life and hence attracted. Thus, green brands should seek ways to elicit positive cognitive and emotional responses from consumers to increase their brand trust.

### Limitations and Future Directions

In spite of the aforementioned contributions, limitations also abound in the scope of the research. First, the study only investigated eight dimensions of the three means of persuasion and did not examine which appeal is the most important in determining brand sincerity. Some prior studies argue that price as a product feature should be included in logos (Chu et al., 2014; Yang et al., 2018). It is also relevant to the perceived economic value (Zauner et al., 2015). However, the study did not consider it a factor in logos because a brand story is more about how a brand is initiated and only the green features of products will be included (Huang and Guo, 2021). Prior studies also found that pathos in a story is more likable than logos and ethos (Samuel-Azran et al., 2015), and ethos represented by customer reviews will be a more important information appeal for Airbnb users to book (Han et al., 2019). Therefore, future research could continue the study with a wider range of dimensions of rhetoric and explore which means of persuasion have the highest influence on brand sincerity or other brand personalities.

Second, although the study presents the demographic information of the participants, it only serves as a general background, characterizing the research. Gender has always been regarded as a critical issue in virtual behavior studies. Men and women diverge yawningly in terms of information processing patterns (Richard et al., 2010), which implicates that the perceived value of a green brand story may vary across genders. Therefore, future research should investigate the role of gender or other descriptive measures in moderating the relationship of the studied constructs. Finally, the data were collected from participants in China; the proposed model should be further tested by gathering samples from users of other nationalities, owing to the fact that cultural disparities may exert an impact on the behavior of consumers (Lin et al., 2009). Therefore, future studies can probe if cultural differences will moderate the studied effect.

## Data Availability Statement

The original contributions presented in the study are included in the article/Supplementary Material, further inquiries can be directed to the corresponding author/s.

## Author Contributions

CH was responsible for theoretical derivation, research design, and manuscript writing. SZ was responsible for literature review, experimental operation, and data analysis. ZL and JG were in charge of data collection and manuscript proofreading. All authors contributed to the article and approved the submitted version.

## Conflict of Interest

The authors declare that the research was conducted in the absence of any commercial or financial relationships that could be construed as a potential conflict of interest.

## Publisher’s Note

All claims expressed in this article are solely those of the authors and do not necessarily represent those of their affiliated organizations, or those of the publisher, the editors and the reviewers. Any product that may be evaluated in this article, or claim that may be made by its manufacturer, is not guaranteed or endorsed by the publisher.
