# Supplementary material for: Creating a Sincere Sustainable Brand: The Application of Aristotle’s Rhetorical Theory to Green Brand Storytelling
Source: Front Psychol. 2022 Jun 2;13:897281. doi: 10.3389/fpsyg.2022.897281 (PMC9202827; doi:10.3389/fpsyg.2022.897281)
Supplement: Supplementary file 1 [file Data_Sheet_1.docx]

Appendix

Study 1

Green brand story with three means of persuasion

I SEE was founded in Ningbo, Zhejiang in 2002. The founder, Chen Deshan was an antique glass bottle collector who was obsessed with things made of glass. One day when he was walking along a seashore, he saw many shining glass fragments lying in the sand. They may hurt those walk barefoot, he thought, “Why not making more recyclable and unbreakable glass?” Then I SEE was born. In the past 19 years, we grew from a glass supplier to a high-end glassware producer. Toughened in the manufacturing process, our glass is six times stronger than regular glass. You would not have to worry small children face hazard of broken glass. Our recycled glass may look fragile but is mighty, which can reduce related water pollution by 50%. Dedicated to protecting the oceans that look mighty but is fragile, we have received Sustainable Product and Materials Certification in China. We sincerely believe you will find “strength” in our glass.

Contact us: S#150, 5th Xining Road, IS Factory, Ningbo, Zhejiang, China. Tel: 86-574-66506351. Fax: 86-574-66511422. Email: I SEE@sina.com

Green brand story without three means of persuasion

I SEE was founded in Ningbo, Zhejiang in 2002. The founder, Chen Deshan was an antique glass bottle collector who was obsessed with things made of glass. Once on a beach vocation, when he was walking along a seashore, he saw many glass fragments scattered in the sand here and there. They were shining in the sunlight and may hurt those walk barefoot. An idea suddenly came to him: why not making more recyclable and unbreakable glass? Then I SEE was born. Initially we were just committed to supplying high quality glass to beverage producers and cosmetic manufacturers. Gradually with the accumulation of more experience and the advantage of material resources, we started to produce custom of high-end glassware products. Toughened in the manufacturing process, our glass is much stronger than regular glass. Our recycled glass can reduce related air pollution and related water pollution, dedicated to protecting the oceans. As a sophisticated glass maker, we find great pleasure in providing high quality products and backing you with sales and customer service with all our hearts.

Study 3

Green brand story with three means of persuasion

I initiated Fabrilliant in Suzhou in 2018. One morning in August, I put on a new shirt to go climbing with a friend. It’s hot that day and my sweat was sweating. About half-way up the mountain, I had rash around my neck because of the fibers in my new shirt. I was embarrassed because I had to give up the climbing. This shirt “irritated” me, so I decided to create an apparel brand using natural fibers. The road to success was often full of obstacles, but I didn’t give up this time. The elusive weather drove Fabrilliant to use recycled cotton to reduce carbon footprint. However, many recycled clothes were poly-cotton blends, and there was no technology in China to tease apart the two twisted fibers. Fortunately, our expert got the news that Sweden had developed an “eco-machine” to separate the cotton and polyester. We immediately introduced this machine and made clothes recycling a reality. Mixed with 22% recycled cotton and 78% organic cotton, our clothes are soft and skin-friendly, which are certified by the Global Organic Textile Standard (GOTS). We can promise that no matter where you start, Fabrilliant will be a good “company”.

Contact us: S#198, 3rd Wuyu Road, Suzhou Industrial Zone, Suzhou, Jiangsu, China. Tel: 86-512-63506352. Fax: 86-512-63517444. Email: fabrilliant@sina.com

Green brand story without three means of persuasion

I initiated Fabrilliant in Suzhou in 2018. One Sunday morning in August, I put on a new shirt to go climbing with a new friend I met online. It’s hot that day and my sweat was sweating it’s that bad. About half-way up the mountain, I had red rash on my face and around my neck because of the fibers in my new shirt. I was embarrassed as hell, because I had to go back to see a doctor immediately. This unpleasant experience made me decide to create a brand of clothes made with natural fibers. Success was challenging. We always met with failures, but I didn’t give up this time. I have to firstly learn some specialty knowledge of textile, and then to overcome difficulties one by one. The summer weather was becoming elusive, so Fabrilliant decided to use recycled cotton to reduce carbon footprint. However, many recycled clothes were poly-cotton blends, and there was no technology in China to tease apart the two twisted fibers at that time. We seemed to be stuck in a dilemma. Fortunately, we then were soon excited by a good news that Sweden had developed an “eco-machine” to separate the cotton and polyester. We immediately introduced this machine and made clothes recycling a reality. We can promise that no matter where you start, Fabrilliant will always accompany you there.
